# Implication of vascular endothelial growth factor A and C in revealing diagnostic lymphangiogenic markers in node-positive bladder cancer

**DOI:** 10.18632/oncotarget.15669

**Published:** 2017-02-24

**Authors:** Cédric Poyet, Linto Thomas, Tobias M Benoit, David Aquino Delmo, Laura Luberto, Irina Banzola, Michèle S Günthart, Giovanni Sais, Daniel Eberli, Tullio Sulser, Maurizio Provenzano

**Affiliations:** ^1^ Oncology Research Unit, Department of Urology and Division of Surgical Research, University Hospital of Zurich, Zurich, Switzerland

**Keywords:** lymphangiogenesis, bladder cancer, diagnosis, VEGF-A, SLP-76

## Abstract

Several lymphangiogenic factors, such as vascular endothelial growth factors (VEGFs), have been found to drive the development of lymphatic metastasis in bladder cancer (BCa).

Here, we have analyzed the gene expression of lymphangiogenic factors in tissue specimens from 12 non-muscle invasive bladder cancers (NMIBC) and 11 muscle invasive bladder cancers (MIBC), considering tumor and tumor-adjacent normal bladder areas obtained from the same organs. We then compared the results observed in patients with those obtained after treating human primary bladder microvascular endothelial cells (MEC) with either direct stimulation with VEGF-A or VEGF-C or by co-culturing (trans-well assay) MEC with bladder cancer cell lines varying in VEGF-A and VEGF-C production based on tumor grade.

The genes of three markers of lymphatic endothelial commitment and development (PDPN, LYVE-1 and SLP-76) were significantly overexpressed in tissues of MIBC patients showing positive lymphovascular invasion (LVI+), lymph node metastasis (Ln+) and tumor progression. Their expression was also significantly enhanced either after direct stimulation of MEC by VEGF-A and VEGF-C or in the trans-well assay with each bladder cancer cell line.

SLP-76 showed the highest gene expression. Both VEGF-A and VEGF-C also enhanced the expression of SLP-76 protein in MEC. However, a correlation between increase of SLP-76 gene expression and the ability of MEC to migrate could only be seen after induction by VEGF-C.

The significant expression of SLP-76 in LVI+/Ln+ progressive MIBC and its overexpression in MEC after VEGF-A and VEGF-C stimulation suggest the need to develop this regulator of developmental lymphangiogenesis as a diagnostic tool in BCa.

## INTRODUCTION

Bladder cancer (BCa) is the most frequent cancer in the urogenital tract in both sexes, with an estimated 77,000 new cancer cases and approximately 16,000 cancer deaths in the United States in 2016 [1, 2]. BCa is generally either classified as non-muscle invasive (NMIBC; ∼80%), hereafter referred to as superficial, or as muscle-invasive cancer (MIBC), based on the natural history of the tumors. Among the superficial tumors, 50%–70% recur after transurethral resection (high-risk NMIBC) [3] and 10%–20% thereof show progression to MIBC [4]. Due to the worse prognosis of MIBC, there is great interest in identifying markers that can diagnose superficial cancers with an increased risk of progression [5, 6]. Approximately 25% of patients undergoing radical cystectomy for MIBC show lymph node metastases (Ln+) [7] and Ln+ are one of the most important predictors of cancer patient outcome [8, 9]. Therefore, there is great interest in studying the role of lymphangiogenic growth factors in patients bearing BCa with an increased risk of disease progression (Ln+) [10]. Vascular endothelial growth factors (VEGFs), in particular VEGF-C and VEGF-D, have been described as regulators of lymphangiogenesis in BCa [11] and the expression of VEGF-C was found to correlate with pelvic lymph node metastases and poor prognosis in this malignancy [12]. In our setting, high VEGF-D serum levels were shown to predict lymph node metastasis in patients with invasive BCa [13]. VEGFs have been proposed to determine a state of cancer invasion and dissemination by increasing the expression of factors involved in lymphatic vessel development and maturation [14]. The lymphatic vessel density (LVD) was found to strongly correlate with lymph node metastases in patients with invasive BCa [15].

Despite above investigations, the synergic role of VEGFs in eliciting a peculiar lymphoangiogenic profile in BCa with a high risk of lymphatic tumor spreading has not been entertained yet. It is thus of utmost importance to identify lymphangiogenic markers in node-positive BCa for diagnostic purposes and to redirect treatment options [16] from targeting the VEGF/VEGFR pathways [17].

## RESULTS

### Gene expression analysis of lymphangiogenic factors in tumor specimens of LVI+/Ln+ MIBC patients

The gene expression of factors involved in tumor lymphangiogenesis was evaluated in tumor tissues of 23 BCa patients and in as many tumor-adjacent normal bladder tissues. The following genes were tested: VEGF-A, C, and D and their tyrosine kinase receptors (VEGFR-2 and VEGFR-3); podoplanin (PDPN) as a specific lymphatic vessel marker; lymphatic vessel endothelial receptor 1 (LYVE-1) as marker for lymphovascular invasion; the C-C chemokine receptor type 7 (CCR7) as a chemoattractant for endothelial cells; angiopoietin-2 (Ang-2) as a modulator of proliferation and migration of endothelial cells and SLP-76 for microenvironmental involvement in lymphangiogenesis [18–20]. Among the 23 patients analyzed in this study, 11 (48%) were <70 yrs and 12 (52%) were ≥70 yrs; 16 (70%) were male and 7 (30%) female. Out of the 23 patients, 12 (52%) and 11 (48%) were diagnosed with non-muscle invasive bladder cancer (NMIBC) and muscle invasive bladder cancer (MIBC), respectively (see Material and Methods). All 23 BCa were shown to be of high histologic grade. Eight out of 23 patients (35%) had lymphovascular invasion (LVI+) and 6 out of 23 (26%) were diagnosed as lymph node positive (Ln+). Seven out of 23 patients (30%) showed tumor progression (Table 1). A significant association between lymphovascular invasion (LVI+) and lymph node metastasis (Ln+) was observed (p < 0.001). Both LVI+ and Ln+ associated with age at diagnosis (p < 0.01). Six out of the 7 patients with tumor progression were LVI+/Ln+ (LVI+, p < 0.01 and Ln+, p < 0.001) (Table 1).

**Table 1:** Distribution of clinicopathologic characteristics of patients (n = 23) who underwent radical cystectomy for BCa and their association with lymphovascular invasion (LVI) and lymph node staging (Ln)

Variable	Categorization	n analyzable	%	association^c^			
				LVI+		Ln+	
Age at diagnosis							
	<70 years	11	48	7		6	
	≥70 years	12	52	1	**<0.01**	0	**<0.01**
Sex							
	male	16	70	5		3	
	female	7	30	3	0.7	3	0.3
Tumor stage^a^							
	NMIBC	12	52	2		1	
	MIBC	11	48	6	0.09	5	0.07
Histologic grade^b^							
	low grade	0	0	0		0	
	high grade	23	100	8	1	6	1
Lymphovascular invasion (LVI)							
	positive	8	35	-		6	
	no LVI	15	65	-	n.a.	0	**<0.001**
Lymph node staging (Ln)							
	positive	6	26	6		-	
	negative	17	74	2	**<0.001**	-	n.a.
Tumor progression							
	yes	7	30	6		6	
	no	16	70	2	**<0.01**	0	**<0.001**
Cancer death							
	yes	1	4	1		1	
	no	22	96	7	0.3	5	0.3

^a^ Stage grouping according to the European Association of Urology 2016 guidelines [3]

^b^ Grading according to the 2004 WHO classification system

^c^ Fisher's exact test, 2-sided

**Figure 1:**
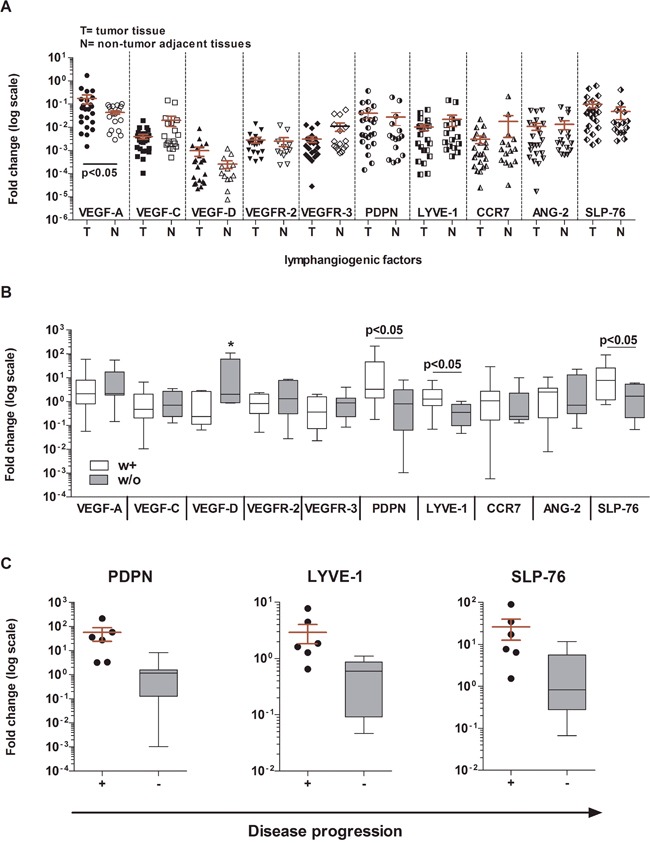
Gene expression analysis of BCa tumor tissues as compared with normal bladder tissues **A**. Gene expression of 10 factors involved in tumor lymphangiogenesis in BCa tissues (T) and in normal bladder tissues obtained from the same patients (N). No significant differences were found, except for the higher expression of VEGF-A in tumor tissues than in normal tissues (Wilcoxon matched-paired test, p < 0.05). The 2^–ΔΔCt^ method was used to compute the gene expression fold change in all factors, setting β-actin gene expression equal to 1. All values ≤10^−7^ were considered undetectable. **B**. Gene expression of the same 10 factors in patients stratified according to clinicopathologic characteristics (white boxes: at least one or w+; n = 12: gray boxes: without or w/o; n = 11). Three genes were expressed at significantly higher levels in w+ patients than in w/o patients: PDPN p < 0.05; LYVE-1 p < 0.05; SLP-76 p < 0.05 (Mann Whitney U test). For VEGF-D (*) medians are not significantly different. **C**. PDPN, LYVE-1, and SLP-76 were overexpressed in LVI+/Ln+ MIBC patients (n = 6) showing tumor progression (scattered dot plot) as compared with those NMIBC patients with no progression (n = 12; gray boxes) (Mann Whitney U test; p < 0.01). For each test performed, a 2^–ΔΔCt^ method was used to compute gene expression fold change in all factors in tumor tissues relative to their gene expression in paired normal tissues. Beta-actin was used as the reference gene. All values ≤10^−7^ were considered undetectable.

The expression of all factors in tumor tissues was not significantly different from that in paired tumor-adjacent normal tissues, except for VEGF-A (p < 0.05) (Figure 1A) and not significantly related to the patient's clinical information. In contrast, the fold change in gene expression of lymphangiogenic factors observed in tumor tissues, relative to those in tumor-adjacent normal tissues as reference genes, showed a clear contribution of the tumor-induced lymphatic vasculature to BCa progression. Indeed, the expression of three factors mainly involved in lymphatic commitment and development, PDPN (p < 0.05), LYVE-1 (p < 0.05) and SLP-76 (p < 0.05), was significantly higher in patients *with* at least one clinicopathological parameter (w+; n = 12), compared with patients *without* these properties (w/o; n = 11; LVI-/Ln- NMIBC) (Figure 1B, Table 2). Even more robust was the significant overexpression of PDPN, LYVE-1 and SLP-76 (p < 0.01) when gene expression fold changes in the 6 LVI+/Ln+ MIBC patients (Pt 6, 7, 10, 12, 18, 20) showing tumor progression and in the 11 LVI-/Ln- NMIBC patients (Pt 1, 3, 4, 5, 9, 11, 16, 17, 21, 22, 23) without progression were compared (Figure 1C, Table 2).

**Table 2:** Distribution of clinicopathological parameters among patients

Patients	Tumor stage	Lympho-vascular invasion	Positive lymph node	Progression	Cancer death	Parameters
**n = 23**	**MIBC= 1, NMIBC = 0**	**yes = 1, no = 0**	**yes = 1, no = 0**	**yes = 1, no = 0**	**yes = 1, no = 0**	**yes = 1, no = 0**
*Pt 1*	0	0	0	0	0	0
Pt 2	**1**	0	0	**1**	0	**1** (2)
*Pt 3*	0	0	0	0	0	0
*Pt 4*	0	0	0	0	0	0
*Pt 5*	0	0	0	0	0	0
**Pt 6**	**1**	**1**	**1**	**1**	0	**1** (4)
**Pt 7**	**1**	**1**	**1**	**1**	0	**1** (4)
Pt 8	**1**	0	0	0	0	**1** (1)
*Pt 9*	0	0	0	0	0	0
**Pt 10**	**1**	**1**	**1**	**1**	**1**	**1** (5)
*Pt 11*	0	0	0	0	0	0
**Pt 12**	**1**	**1**	**1**	**1**	0	**1** (4)
Pt 13	**1**	0	0	0	0	**1** (1)
Pt 14	**1**	**1**	0	0	0	**1** (2)
Pt 15	**1**	0	0	0	0	**1** (1)
*Pt 16*	0	0	0	0	0	0
*Pt 17*	0	0	0	0	0	0
**Pt 18**	**1**	**1**	**1**	**1**	0	**1** (4)
Pt 19	0	**1**	0	0	0	**1** (1)
**Pt 20**	**1**	**1**	**1**	**1**	0	**1** (4)
*Pt 21*	0	0	0	0	0	0
*Pt 22*	0	0	0	0	0	0
*Pt 23*	0	0	0	0	0	0
**with (w+)**	**11**	**8**	**6**	**7**	**1**	**12**
**without (w/o)**	**12**	**15**	**17**	**16**	**22**	**11**

In bold: LVI+/Ln+ MIBC patients with progression (n = 6)

In italics: LVI-/Ln- NMIBC patients without progression (n = 11)

### *In vitro* crosstalk between MEC and BCa cells confirmed the gene expression profile observed *ex vivo* in patients

To mimic the *in vivo* cross talk between BCa and endothelial cells and to reconfirm the observations made *ex vivo* in patients, we decided to prime MEC with tumor derived soluble factors released by three human BCa cell lines (RT4, HTB-9 and T24) with different tumor grading using a trans-well co-culture assay. The human uroepithelial cell line UROtsa was used as control. Gene expression analysis of lymphatic vessel markers and vessel-forming factors, such as lymphatic vessel endothelial receptor 1 (LYVE-1), angiopoietin-2 (Ang2), podoplanin (PDPN), chemokine ligand 21 (CCL21), prospero-related homeobox 1 (PROX-1), forkhead box 2 (Fox-2), ephrin-B2 (EFNB-2), vascular endothelial growth factor receptor 2 (VEGFR-2) and 3 (VEGFR-3) [18–20], and CD31 [21] was carried out. Due to the relevant overexpression in patients, SLP-76 was also tested. A constitutive gene expression analysis was performed to compute fold changes. Hence, MEC were cultured without stimuli for 48 h. Eight of the tested factors showed and maintained high gene transcription over time compared with CD31, while two (CCL21 and SLP-76) were weakly expressed (Supplementary Figure 1).

**Figure 2:**
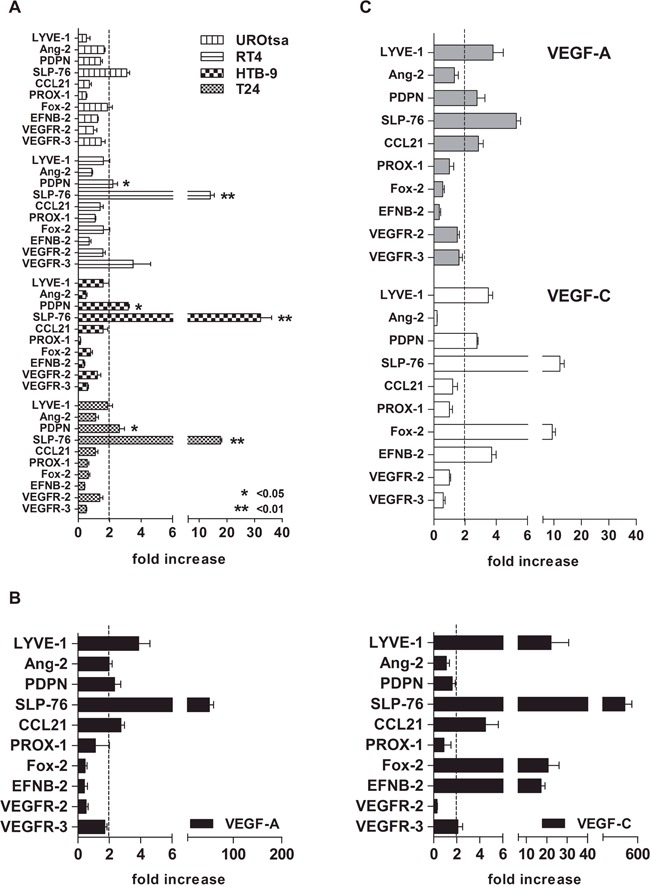
Gene expression profile of lymphatic vessel-forming factors and VEGFRs by cell line trans-well assay **A**. MEC at 80% confluence were co-cultured for 24 h with either BCa cell line (test) or UROtsa cells (control) previously grown in co-culture inserts. A significant overexpression of two genes (PDPN and SLP-76) over the 2-fold baseline (arbitrary cut-off) was observed in all three BCa cell lines, as compared with control (UROtsa; * p < 0.05 and ** p < 0.01, respectively). **B**. The cells showed different factor and receptor profiles upon VEGF-A and VEGF-C stimulation at the standard concentration of 50 ng/ml with a stronger Fox-2 and EFNB-2 increase (20-fold) after VEGF-C stimulation, as compared to that after VEGF-A stimulation (p < 0.001, Mann Whitney U test). **C**. Although to a lesser extent (approximately 10-fold less), the factor and receptor profile upon VEGF-A and VEGF-C stimulation at concentrations of 17 ng/ml and 2 ng/ml, respectively (“established” concentration; see Table 2 for calculation) was similar to the pattern induced at standard concentrations. For both tests (standard and established concentrations), the 2^–ΔΔCt^ method was used. Fold increase was calculated as the gene expression fold change in all factors and receptors above their constitutive expression (2-fold baseline as cut-off). CD31 was used as the reference gene. Error bars represent the mean ± SD of three replicates.

The three MEC/RT4, MEC/HTB-9, and MEC/T24 co-cultures showed a similar gene expression profile of the factors and receptors studied. Particularly, PDPN gene expression levels were significantly increased above the 2-fold threshold in these three co-cultures compared with MEC/UROtsa: (MEC/RT4 2.2-fold ± 0.5; MEC/HTB-9 3.2-fold ± 0.1; MEC/T24 2.6-fold ± 0.6; MEC/UROtsa 1.4-fold ± 0.2; p < 0.05), while SLP-76 gene levels were expressed at significantly higher levels over the 2-fold baseline (MEC/RT4 14-fold ± 2.4; MEC/HTB-9 32.2-fold ± 6.8; MEC/T24 17.7-fold ± 0.8; MEC/UROtsa 3.1-fold ± 0.3; p < 0.01). In contrast, LYVE-1 maintained non-significant gene expression slightly below the 2-fold baseline for all co-cultures compared with MEC/UROtsa (MEC/RT4 1.6-fold ± 0.5; MEC/HTB-9 1.7-fold ± 0.7; MEC/T24 1.96-fold ± 0.5; MEC/UROtsa 1.3-fold ± 0.2; Figure 2A).

### The effect of a direct VEGF-A and VEGF-C induction of MEC at concentrations similar to that produced by BCa cell lines

To attribute the genetic profile observed after culturing MEC with BCa cell lines to VEGF activity, we performed a direct stimulation of MEC with both VEGF-A and VEGF-C. Concentrations were established by computing fold changes in gene expression and protein release at a constitutive level over 72 h of each tumor cell line used in this study based on their tumor grade. UROtsa cells were used as a control (supplementary material). We noted that VEGF-C was produced at a significantly higher level than VEGF-A by the two high-grade BCa cell lines HTB-9 and T24 (p < 0.0001 and p < 0.001, respectively), whereas only the low-grade cell line RT4 produced significant amounts of VEGF-A (p < 0.01). The expression of VEGF-D was negligible and non-significant in all cell lines tested (Supplementary Figure 2A and 2B). In keeping with gene expression data, the RT4 cells produced the highest amount of VEGF-A protein among the three cancer cell lines tested (p < 0.01) and compared with the UROtsa cell line (p < 0.05). In contrast, there was a significant release of VEGF-C protein by all cell lines, although to different extents (p < 0.05; Supplementary Figure 2C).

The stimulation of MEC with either standard (50 ng/ml) or established concentrations of VEGF-A (17 ng/ml) and VEGF-C (2 ng/ml; supplementary material; Table 3) generated a gene profile similar to the one observed after performing the trans-well assay, except for the overexpression of Fox-2 and EFNB-2 upon direct stimulation with VEGF-C. The increase in gene transcript of PDPN, LYVE-1, and SLP-76 over the 2-fold baseline was observed upon either VEGF-A or VEGF-C stimulation at either standard (Figure 2B) or established concentrations (Figure 2C).

**Table 3:** Established concentrations for VEGF-A and VEGF-C

Amount of VEGFs: pg/cell *per* size of growth area									
									mean
Factor	Cell line	150 cm^2^	75 cm^2^		25 cm^2^		9.6 cm^2^		pg/cell*
VEGF-A	RT4	2,02^−1^	1.49^−1^		1.31^−1^		1,07^−1^		**1.47^−1^**
VEGF-C	RT4	7.32^−3^	8,01^−3^		8,11^−3^		1,05^−2^		**8.50^−3^**
VEGF-C	HTB-9	1,09^−2^	1.42^−2^		1.25^−2^		2.39^−2^		**1.54^−2^**
VEGF-C	T24	2,03^−2^	1.82^−2^		1.38^−2^		1.96^−2^		**1.80^−2^**
*Normalized amount									
**Calculation of established concentrations (ng)**										
Factor	Cell line	n of cells*		pg/cell		total concentration in ng				
VEGF-A	RT4	117206		**1.47^−1^**		1.73^4^		**17 ng**		
VEGF-C	RT4	117206		**8.50^−3^**		9.96^2^		**1 ng**		
VEGF-C	HTB-9	132685		**1.54^−2^**		2,04^3^		**2 ng**		
VEGF-C	T24	163042		**1.80^−2^**		2.93^3^		**3 ng**		
average				**1.39^−2^**		1.99^3^		**2 ng**		
*insert size= 4.2 cm^2^										

### The effect of VEGF-C stimulation on SLP-76 expression in MEC

**Figure 3:**
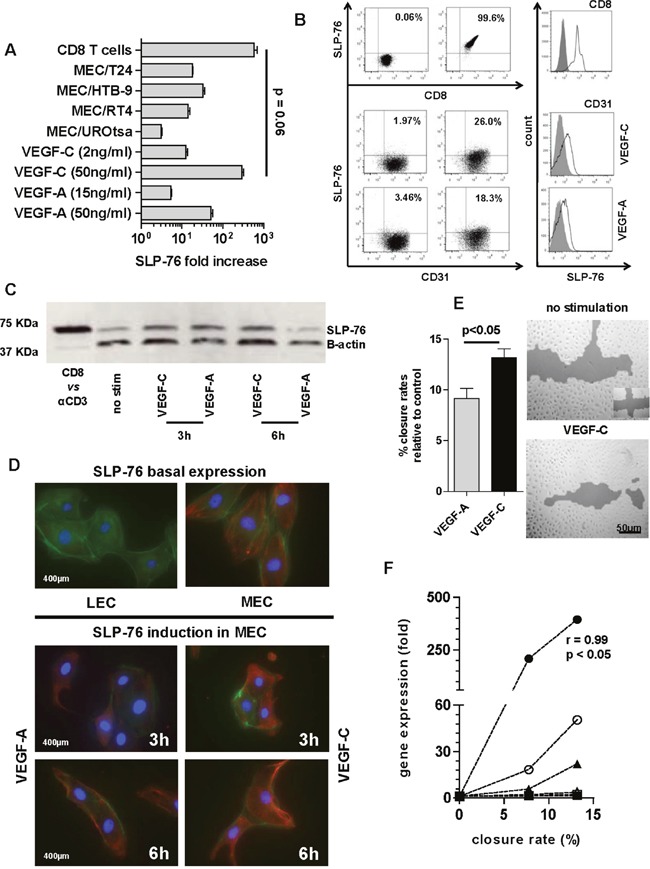
SLP-76 expression and production in MEC **A**. The SLP-76 gene expression upon VEGF stimulation (at either concentration) and under bladder cell line trans-well assay was compared with SLP-76 gene expression elicited in anti-CD3 treated CD8 T cells (positive control). SLP-76-fold increase after direct MEC stimulation or priming with cell lines was calculated as gene expression 2-fold over its constitutive expression (arbitrary cut-off). SLP-76-fold increase in anti CD3 treated CD8 T cells was calculated as gene expression 2-fold over SLP-76 gene expression in non-stimulated CD8. Error bars represent the mean ± SD of three replicates. **B**. Representative fluorescent-activated cell sorting (FACS) dot plot analysis of *in vitro* SLP-76 intracellular staining in anti-CD3 treated CD8 T cells (upper right panel) and VEGF-C (2 ng/ml medium right panel) or VEGF-A (17 ng/ml lower right panel) stimulated CD31+ MEC, as compared with isotype staining (all left panels). Overlaid histograms refer to mean fluorescent intensity (MFI) of *in vitro* SLP-76 intracellular staining of CD8 T cells upon anti-CD3 induction (above) or MEC after VEGF-C (middle) or VEGF–A (below) stimulation compared with isotype staining. **C**. Western blot showing SLP-76 expression in MEC stimulated with either VEGF-A or VEGF-C at 50 ng/ml, over 3 and 6 h, as compared with SLP-76 expression in human CD8 T cells treated with anti-CD3 mAb (CD8 *vs* αCD3). **D**. A representative image by fluorescence microscopy (scale bar 400 μm) of MEC after 3 and 6 h of stimulation with either VEGF-A or VEGF-C (50 ng/ml). The SLP-76 basal staining (no stimulation) for both cell lines (MEC right and LEC left) is also reported. MEC were immunostained for SLP-76 (Cy-3; red), cytoskeleton (phalloidin; green), and nucleus (DAPI; blue). **E**. MEC migration was assessed by a scratch wound healing assay, as shown in one representative picture (scale bar 50 μm) by confocal microscopy after 24 h of VEGF-C stimulation at the standard concentration. Cells were cultured in 6-well plates and allowed to reach 80% confluence before scratching vertically and horizontally (see symbol bottom right in the upper panel). Histograms represent migration potential of cells based on the quantification of empty area (% closure rate) as compared with time 0 (100% empty area) for VEGF-A (gray bar) or VEGF-C (black bar) (p < 0.05; Mann Whitney U test). Error bars represent the mean ± SD of three replicates. **F**. MEC closure rates were plotted against gene expression of PDPN, LYVE-1, and SLP-76 after VEGF stimulation. The 3 time points tested (0, 16, and 24 h) were reported. SLP-76 upon VEGF-C stimulation (black circle) showed a significant linear correlation between the increase in gene expression and the percentage closure rate of MEC (r = 0.99, p < 0.05; Pearson correlation coefficient), as compared with VEGF-A stimulation (open circle) and with both other factors upon both stimulations (PDPN; black and open square) and LYVE-1; black and open triangle).

Among the genes overexpressed after MEC induction, SLP-76 showed the highest expression. Notably, direct MEC stimulation with VEGF-A and VEGF-C at either standard (VEGF-A–50 ng/ml 50.54-fold ± 9.1; VEGF-C–50 ng/ml 393.8-fold ± 109) or established concentrations (VEGF-A–15 mg/ml 5.29-fold ± 0.5; VEGF-C–2 ng/ml 12.16-fold ± 2.7) induced a fold change in SLP-76 gene expression similar to that elicited by co-culturing MEC with either BCa cell line. Overall, VEGF-C at the standard concentration of 50 ng/ml elicited the highest SLP-76 amount, similarly to that induced by anti-CD3 treated CD8 T cells (p = 0.06) (Figure 3A).

The SLP-76 gene overexpression observed by co-culturing MEC with BCa cells encouraged us to also investigate SLP-76 protein secretion in these cells under VEGF induction. The analysis was carried out by using 50 ng/ml of VEGF-A and VEGF-C, as no relevant protein expression of SLP-76 was detectable after stimulation with established concentrations of growth factors compared with SLP-76 expression in anti-CD3 induced human CD8 T cells (Figure 3B). Due to the specific cell source used (MEC) in this study, we also tested human dermal lymphatic endothelial cells from juvenile foreskin (HDLEC, hereafter LEC), which are reported not to express SLP-76. As expected, we noted that the constitutive gene expression of SLP-76 in LEC was completely absent and that the induced gene expression upon stimulation (either direct or by trans-well assay) was negligible (Ct values below the reliable limit of detection; data not shown). SLP-76 protein production was then analyzed in both MEC and LEC after 3, 6 and 12 h of VEGF stimulation by both western blot and immunofluorescence (IF) assays. In western blot analysis, we observed an increase in SLP-76 protein production in MEC 3 h after VEGF-A and VEFG-C stimulation, compared with the basal expression of SLP-76 in non-stimulated MEC (Figure 3C). The amount of SLP-76 was maintained 6 h after stimulation with VEGF-C but was drastically reduced 6 h after stimulation with VEGF-A (Figure 3C). The SLP-76 protein production and rate of concentration after VEGF stimulation, as detected by western blot assay, was confirmed by IF assay. Notably, a longer induction with VEGF-C correlated with a change in MEC morphology (Figure 3D). In addition, when performing a migration assay (wound healing assay) of MEC stimulated over 24 h with either VEGF-A or VEGF-C at a concentration of 50 ng/ml, a higher migration capacity of MEC (higher closure rate) was particularly observed after stimulation with VEGF-C (p < 0.05; Figure 3E). Finally, when plotting MEC migration activity against VEGF-specific gene upregulation of PDPN, LYVE-1, and SLP-76, we observed a significant correlation between increment of gene molecules and an increase in closure rates only for SLP-76 after stimulation with VEGF-C (p < 0.01; Figure 3F). In keeping with gene expression analysis, we could not find SLP-76 expression in LEC, either constitutively or upon induction, as documented by IF in LEC showing basal SLP-76 expression (Figure 3D).

## DISCUSSION

As a solid malignancy, BCa depends on lymphangiogenesis for tumor invasion and dissemination [22]. It has been seen that the expression of VEGFs and their receptor VEGFR-2 was significantly higher in invasive than in non-invasive BCa, and that this finding is associated with disease recurrence [23]. In addition, the same study also showed significantly higher expression of VEGFs in tumor tissue than in the adjacent normal bladder mucosa [23]. The gene profile analysis of lymphangiogenic factors detected in tumor tissues of our group of patients showed no statistically significant difference when compared with tumor-adjacent normal bladder tissues, except for VEGF-A. In contrast, we found significantly higher expression levels of three factors (PDPN, LYVE-1, SLP-76) mainly involved in lymphatic commitment and development when using normal tissues as references and after stratification of patients based on clinical parameters and disease progression.

The co-culture of MEC with each of the BCa cell lines confirmed the significant gene overexpression of two of the three factors (PDPN and SLP-76) observed in LVI+/Ln+ MIBC patients with clinical progression. This increase seemed to be VEGF dependent, because direct stimulation of MEC with VEGF-A and VEGF-C induced a similar gene profile as seen when co-culturing MEC with BCa cell lines with either secreted amount of VEGF-A or VEGF-C.

Data aimed at defining the role of lymphangiogenic growth factors in BCa progression showed that the *in vitro* expression of VEGF-A, VEGF-C, and VEGF-D, as detected in BCa cell lines, varies among histological variants of BCa and correlates with grading. VEGF-C was highly expressed in high-grade tumor cells from invasive bladder cancers while VEGF-A in low-grade tumor cells from a non-invasive papillary carcinoma. Although the role of VEGF-A in tumor angiogenesis is established [24], our findings confirmed VEGF-A as inducer and maintainer of a metastatic status in draining lymph nodes [25, 26] and indicate its involvement (with the latter function) in the progression of NMIBC into MIBC. In fact, patients with metastatic BCa treated with monoclonal antibody (i.e., bevacizumab) targeting VEGF-A showed a better outcome [27].

The increase in gene transcripts of factors involved in lymphatic endothelial cell migration and maturation, such as LYVE-1, PDPN, CCL21, Fox-2, and EFNB-2 by VEGF-C induction was confirmed in MEC. In addition, VEGF-C and to a lesser extent, VEGF-A, but not VEGF-D, could directly enhance the migration potential of MEC. These findings corroborated VEGF-C as a driver of lymphangiogenesis in BCa. In addition, they pointed out the support that VEGF-A provides to synergistically induce the expression of endothelial factors and receptors in MEC. In contrast, our findings could not confirm the predictive role of VEGF-D in BCa progression and disease outcome, as recently observed either locally by others [10] or systemically by us [13]. Rather, the higher expression of VEGF-D in LVI-/Ln- NMIBC patients and its slightly higher expression in low-grade tumor cells, although both lack significance, is controversial. The expression of the VEGF-D gene close to the limit of detection (10^−7^-fold change to CD31) observed in tumor-adjacent normal tissues from LVI-/Ln- NMIBC patients could have led to overestimation of the VEGF-D expression level in this group of patients.

Among all factors tested, SLP-76 showed the highest significantly increased expression in each zco-culture performed. Particularly, VEGF-C elicited the highest fold-change at either established (>10-fold over the baseline) or standard (>300-fold over the baseline) concentration. To validate gene expression data, SLP-76 protein was detected at either constitutive level or after stimulating MEC with VEGF-A and VEGF-C. Finally, SLP-76 expression was strongly correlated with the ability of MEC cells to migrate upon VEGF-C induction.

SLP-76 is a signaling protein whose main role is to drive the separation of blood from lymphatic endothelial cells [28]. It is regularly produced by platelets while the generation of new lymphatic vasculature, mainly sprouting from pre-existing blood vessels, is typically enhanced by PDPN triggering of platelets. Its expression in lymphatic endothelial cells or precursor cells is required for vascular separation during development [29]. Therefore, the significant expression of SLP-76, together with that of LYVE-1 and PDPN, observed in our cohort of patients led us to believe that its role in bladder tumor spreading might be preeminent and deserves further investigation. Our data suggest a robust expression of this signaling protein in bladder microvascular endothelial cells under VEGF-A and VEGF-C induction. It is thus tempting to speculate that VEGF-induced SLP-76 orchestrates the development of new lymphatic vasculature from pre-existing lymphatic vessels in BCa [29]. However, SLP-76 overexpression in MEC is not documented. We are aware that technical problems could generate an artifact at the level of gene expression. Particularly, by normalizing gene data to low constitutive levels, an overestimation can occur. However, in support of our findings, any expression of the SLP-76 gene in MEC at a constitutive level was always above the detection limit (10^−7^-fold change to CD31). In addition, to test the integrity of our investigation, we used as a control LEC in which SLP-76 expression is not expected. We confirmed the absence of SLP-76 in LEC at both gene and protein levels. Moreover, this expression in MEC cannot be attributed to their senescence after increased passages, because all our experiments were performed on cell lines between passage 5 and 9.

In conclusion, the very novelty of this investigation was the expression at high concentration of SLP-76 *ex vivo* in progressive LVI+/Ln+ MIBC and *in vitro* in MEC upon VEGF induction. This finding opens the question of whether SLP-76 produced within the BCa tumor environment could influence cancer lymphangiogenesis and predispose the patient to a worse prognosis.

## MATERIALS AND METHODS

### Patients and tumor classification

Patients diagnosed with aggressive bladder cancer who underwent radical cystectomy and bilateral pelvic lymph node dissection at the University Hospital of Zurich between 2009 and 2013 were consecutively enrolled. For this study, only 23 patients could be included because enough tumor and non-tumor material was provided after routine inspection and diagnostic of the bladder specimen by an expert pathologist. To simplify classification of urinary bladder cancers, we adopted the EAU guidelinehttps://uroweb.org/wp-content/uploads/EAU-Guidelines-Non-muscle-invasive-Bladder-Cancer-2015-v1.pdf. Therefore, all non-invasive papillary carcinomas, carcinoma *in situ* and tumors only invading subepithelial connective tissue were grouped as non-muscle invasive bladder cancer (NMIBC). Tumors showing evidence of invasiveness from superficial muscles to surrounding organs were grouped as muscle invasive bladder cancer (MIBC). All patients provided written informed consent and the study was approved by the Cantonal Ethical Committee of Zurich (KEK-StV-Nr. 02/09).

### Tissue processing

All pathological specimens were processed according to standardized institutional procedures. Immediately after complete removal, the bladder was examined by an experienced pathologist and both tumor and tumor-adjacent tissues were obtained from each patient enrolled. Specimens were cut into small pieces and snap frozen in liquid nitrogen. Then, tissues were ground with a chilled mortar and pestle for quality RNA extraction (Ambion RNA Aqueous Kit, Thermo Fisher Scientific, Switzerland).

### Cell lines

Three human BCa cell lines (HTB-2 or RT4, HTB-9 and HTB-4 or T24) with different tumor grading [30, 31] were obtained from the American Type Culture Collection (ATCC, LGC Standards, USA). The low-grade RT4 cell line was developed from a non-invasive transitional cell papilloma of the bladder. The high-grade HTB-9 and T24 cell lines were developed from primary high invasive carcinomas of the bladder. The human uroepithelial cell line UROtsa was a kind gift from Prof. Scott Garrett, Department of Pathology, University of North Dakota, USA. BCa cell lines were maintained in RPMI medium supplemented with 100 μg kanamycin, 1 mM GlutaMAX, 1 mM sodium pyruvate, 10 mM HEPES and non-essential amino-acids (all from Gibco, Paisley, Scotland), as well as 10% heat-inactivated fetal calf serum (FCS) (Thermo Fisher Scientific, Switzerland). UROtsa cells were maintained in Dulbecco's Modified Eagles Medium (DMEM) composed of a 1:1 mixture of DMEM and Ham's F-12 supplemented with 100 μg kanamycin, 1 mM GlutaMAX, 1 mM sodium pyruvate, 10 mM HEPES and non-essential amino-acids (all from Gibco, Paisley, Scotland) and 10% heat-inactivated FCS (Thermo Fisher Scientific, Switzerland). The gene expression of Uroplakin II (UPII; marker of terminally differentiated urothelium and sensitive marker for urothelial carcinomas [32, 33]) and Maspin (a serine-protease that inhibits tumor growth and metastasis [34]) were tested to confirm the cancer grading and potential invasiveness of the three cancer cell lines used in this study. UPII gene expression was detected in all three cell lines at levels relative to the tumor grade (1000-fold greater in RT4 than in T24). Maspin gene expression was 5-fold higher in RT4 than in T24 and HTB-9 (data not shown). The human primary bladder microvascular endothelial cells (HBdMEC; hereafter MEC) were obtained from Lonza (Basel, Switzerland) while the human dermal lymphatic endothelial cells (juvenile foreskin; HDLEC; hereafter LEC) were obtained from PromoCell (Heidelberg, Germany). Both endothelial cell lines were cultured as follows: flasks or wells were coated with collagen (50 μg/ml in PBS; BD Bioscience, Switzerland) for at least 20 min at room temperature. Cells were then seeded in EBM-2 basal medium (Lonza, Basel, Switzerland) supplemented with 100 μg kanamycin, 1 mM GlutaMAX, and 20% heat-inactivated FCS (Thermo Fisher Scientific, Switzerland), 25 μg/ml of cAMP and 10 μg/ml of Hydrocortisone (Sigma-Aldrich, Switzerland). All experiments were performed with endothelial cells at passage 9 or lower. For MEC/LEC stimulation, either VEGF-A protein (H00007422-Q01, Abnova, Walnut, CA, USA) or VEGF-C protein (H00007424-P01, Abnova, Walnut, CA, USA) were used. All cell lines were cultured in saturated humidity at 37 °C, under 5% CO_2_, and 95% air. The number of cells to be plated was adjusted according to cell size. Three-day culturing was the appropriate time span for all cell lines to reach 90%–100% confluence (harvesting time).

### Trans-well assay

For co-culturing, MEC were first grown in a 24-well plate to reach 80% confluence. Then, cells were co-cultured with either bladder cancer cells lines (MEC/RT4, MEC/HTB-9 or MEC/T24) or the control cell line (MEC/UROtsa), already grown in inserts to avoid direct contact with MEC, for 24 h.

### Gene expression analysis

After cell harvesting, total RNA was extracted from each cell line condition according to the manufacturer's instructions (RNA Aqueous Kit, Thermo Fisher Scientific, Switzerland) and transcribed into cDNA (High Capacity cDNA Reverse Transcriptase Kit, Applied Biosystems Rotkreuz, Switzerland) to perform gene expression by qRT-PCR. Assays were performed with the TaqMan Gene Expression Assay Kit (Applied Biosystems, Rotkreuz, Switzerland) using “on demand” sets of primers and probes for lymphangiogenic factors and endothelial cell receptors. Reactions were run using the Rotor-Gene 3000 (Corbett Life Science, Sydney, Australia) in a final volume of 20 μl/reaction (19 μl TaqMan mix and 1 μl cDNA). To quantify copy numbers of genes, standard curves from each condition of all cell lines were generated using 1:10 cDNA serial dilutions for each gene tested at known concentration (10^8^ to 10^3^). Beta actin and PECAM1 (CD31) were used as housekeeping genes [21]. Ribosomal RNA subunit 18 (rRNA18S) was used to test the integrity of our assay. Normalized data were evaluated as relative quantification by using the 2^–ΔΔCt^ method to compute fold changes among experimental conditions. When indicated, absolute copy numbers of genes were calculated.

### Protein analysis and expression

The production of VEGF-A, VEGF-C and VEGF-D proteins in supernatants of bladder cell lines was analyzed by using either the human VEGF-A enzyme-linked immunosorbent assay (ELISA) Kit (BMS277) or the human VEGF-C ELISA Kit (BMS297; Bender MedSystems, Wien, Austria) according to the manufacturer's instructions. The detection of VEGF-D was analyzed by human VEGF-D ELISA Kit (LS-F27835; LSBio. Seattle, WA, USA). The supernatant was loaded into a 96-well plate, including standard controls. Protein concentrations were measured with an Emax Plus Microplate Reader and analyzed by SoftMax Pro Software (Molecular devices, Sunnyvale, CA, USA).

### Scratch wound healing assay

Endothelial cells were seeded in triplicate in 24-well plates and grown until confluent. Cells were then incubated overnight in serum-reduced medium containing 1% FBS. Identical scratches (two crosses were scratched in each well) were generated with a 200-μl sterile pipette tip. Any cellular debris was removed by washing with PBS. Serum-reduced medium was replaced and cells were instantly centre-imaged at 5× magnification, using a Zeiss Axiovert 200 M microscope equipped with a Zeiss AxioCam MRm camera with maximum contrast (Carl Zeiss AG, Feldbach, Switzerland, software: Axio Vision Rel. 4.7). For cell stimulation, either VEGF-A or VEGF-C protein were added to the wells at a final concentration of 50 ng/ml. After 24 h, the medium was replaced with PBS and the closure of the scratch by migrating cells was analyzed. Data were evaluated using the Tscratch Software with default parameter settings.

### Flow cytometry

Stimulated or non-stimulated endothelial cells were stained extracellularly with anti-CD31-fluorescin isothiocyanate (FITC) and intracellularly (either before or after stimulation) with anti-SLP-76 allophycocyanin (APC) antibodies (BD Biosciences, Allschwil, Switzerland). As control, stimulated (anti-CD3) or non-stimulated enriched human CD8+ T cells (MACS cell separation, Miltenyi Bergisch Gladbach, Germany) were stained extracellularly with anti-CD8-FITC and intracellularly with anti-SLP-76 APC antibodies (BD Biosciences, Allschwil, Switzerland). Data were acquired on a LSR II Fortessa flow cytometer equipped with FacsDIVA software (BD Biosciences, Allschwil, Switzerland).

### Western blot

Endothelial cells were stimulated with either VEGF-A or VEGF-C at a final concentration of 50 ng/ml for 3, 6, and 12 h. Lysates of VEGF-treated and non-treated cells were prepared by a 1 h incubation on ice with 100 μl of M-PER Mammalian Protein Extraction Reagent and a cocktail of protease inhibitors (Halt Protease Inhibitor Cocktail, Thermo Scientific, Waltham, MA, USA), and subsequent disruption by sonication. After centrifugation for 20 min, protein concentrations were determined in supernatants by a standard Bradford assay. Equal amounts of protein (30 μg) were loaded and separated on an SDS-PAGE gel. After transfer to nitrocellulose membranes, proteins were incubated with the primary antibody SLP-76 (Santa Cruz Biotechnology, Dallas, Texas, USA) overnight at 4 °C and subsequently with a secondary antibody for 1 h, developed with Armersham ECL Western Blotting Detection Reagent (GE Healthcare, Waukesha, WI, USA) and finally, exposed to film. Beta-actin (Abcam, Cambridge, UK) was used as the control gene.

### Immunofluorescence

Endothelial cells were cultured in Lab-tek chambers and allowed to grow until they reached approximately 80% confluence. Stimulations with either VEGF-A or VEGF-C were performed at standard concentrations and times as described above. Cells were fixed with 4% paraformaldehyde for 10 min at room temperature, permeabilized with 1 ml 0.5% Triton X-100 and blocked with 1% BSA. Cells were then incubated for 1 h with the primary antibody SLP-76 (Santa Cruz Biotechnology Dallas, Texas, USA) and overnight at 4°C with the Cy3-conjugated secondary antibody. Dapi was used for staining of nuclei and FITC-conjugated phalloidin for the cytoskeleton. Immunofluorescence images were digitally recorded with a Leica CTR6000 microscope (Leica, Heerbrugg, Switzerland).

### Statistical analysis

Statistical analysis was performed with GraphPad Prism (v5.1) and SAS/STAT (v9.1). The data were reported as mean ± standard error (SE) with median values and ranges where appropriate. Distributions of categorical markers were analyzed by Fisher's exact test. When comparing numerical markers, non-parametric tests, such as the Mann-Whitney U test, Wilcoxon signed rank test and Kruskal-Wallis test were used. The Pearson correlation coefficient was used to measure the strength of linear associations. Differences were considered significant at p < 0.05 (CI 95%).

## SUPPLEMENTARY MATERIALS METHODS AND FIGURES


