# Supplementary material for: Implication of vascular endothelial growth factor A and C in revealing diagnostic lymphangiogenic markers in node-positive bladder cancer
Source: Oncotarget. 2017 Feb 24;8(13):21871–83. doi: 10.18632/oncotarget.15669 (PMC5400630; doi:10.18632/oncotarget.15669)
Supplement: Supplementary file 1 [file oncotarget-08-21871-s001.pdf]

Implication of vascular endothelial growth factor A and C in revealing diagnostic lymphangiogenic markers in node-positive bladder cancer

SUPPLEMENTARY MATERIALS

Expression of VEGF-A and VEGF-C in BCa cell lines with different tumor grading

VEGF-A, VEGF-C, and VEGF-D gene expression was tested by culturing the three bladder cancer cell lines (RT4, T24, HTB-9) in four differently sized growing areas (150 cm^2, T-150; 75cm^2, T-75; 25cm^2, T-25 and 9.6cm^2, 6-well plate) for 72 h. The normal urothelium cell line (UROtsa) was used as a control. To test the integrity of our investigation and standardize VEGF gene expressions in each of the cell lines tested, the means of the number of bladder cancer cells (RT4, T24, HTB-9) and uroepithelial cells (UROtsa) harvested after 72 h of culturing were correlated with the means of the amount of total RNA extracted after cell harvesting. It was clearly shown that the number of cells significantly were correlated with the amounts of total RNA extracted. Indeed, all cell lines expressed a cell count-dependent decrease in VEGF gene transcripts in all growing areas studied, (r range: 0.98–1; $p < 0.01$), although to a lesser extent in UROtsa when compared with the cancer cell lines (data not shown). The two high-grade BCa cell lines HTB-9 and T24 showed the highest VEGF-C gene expression (HTB-9: $r = 1$; $p < 0.0001$ and T24: $r = 0.98$; $p < 0.001$), whereas the low-grade RT4 cell line showed the highest VEGF-A gene expression, ($r = 0.99$; $p < 0.01$) (Supplementary Figure 2A). To better compute the amount of VEGF-A and VEGF-C in cell lines and to reset differences due to cell morphology and time required to attain confluence (90%–100%), we calculated the number of molecules by dividing the number obtained through the absolute quantification method by the size of growth areas (150 cm^2, 75 cm^2, 25 cm^2, 9.6 cm^2). In accordance with the observation above, the overexpression of VEGF-A by the RT4 cell line was statistically significant among the BCa cell lines tested ($p < 0.01$) and when compared with UROtsa ($p < 0.05$), whereas VEGF-C expression by T24 was statistically significant among the three cells lines ($p < 0.01$) and when compared with UROtsa ($p < 0.05$) (Supplementary Figure 2B).

Supernatants of cell lines cultured under conditions as described above were used to calculate the protein release of VEFG-A, VEGF-C and VEGF-D. Following the same rationale adopted for gene expression analysis, we found that RT4 cells released 3.5-fold and 3-fold more VEGF-A than the amount released by HTB-9 and T24, respectively, and 5-fold more than that released by UROtsa ($p < 0.01$). VEGF-C was released in 6-, 4.5- and 3.5-fold higher amounts by T24, HTB-9, and RT4, respectively, than by UROtsa ($p < 0.01$) (Supplementary Figure 2C). Not worthy of note was the release of VEGF-D by each of the cell lines used (< 5 pg/ml; data not shown).

Due to the significant correlation observed between the amount of VEGF-A and VEGF-C gene transcript and protein release (Supplementary Figure 2D), we calculated their “established” concentration as follows. We multiplied the amount of protein released (pg/ml) for each growth factor by the volume used to culture cells in the four differently sized growing areas (20 ml in 150 cm^2, 10 ml in 75 cm^2, 3.3 ml in 25 cm^2, 1.27 ml in 9.6 cm^2). The protein amount was normalized by dividing that obtained (pg) for each growth factor from each sized growing area by the number of cells grown at 90%–100% confluence in the respective 150 cm^2, 75 cm^2, 25 cm^2, and 9.6 cm^2 wells (pg/cell *per* size of growth area). The final amount of VEGF-A or VEGF-C protein released by single cell of each cell line was calculated as the mean of the normalized amount. Indeed, we could determine 1.47×10^{-1} pg/cell for VEGF-A in RT4 cells and on average 1.39×10^{-2} pg/cell for VEGF-C in all three cancer cell lines (8.5×10^{-3} in RT4, 1.54×10^{-2} in HTB-9, and 1.8×10^{-2} in T24) (Table 2). To obtain “established concentrations” of VEGF-A and VEGF-C, we multiplied such amounts by the number or cells contained in the insert (4.2 cm^2) of the trans-well assay. Therefore, we performed a direct stimulation of MEC with either VEGF-A or VEGF-C at concentrations of 17 ng/ml and 2 ng/ml, respectively.

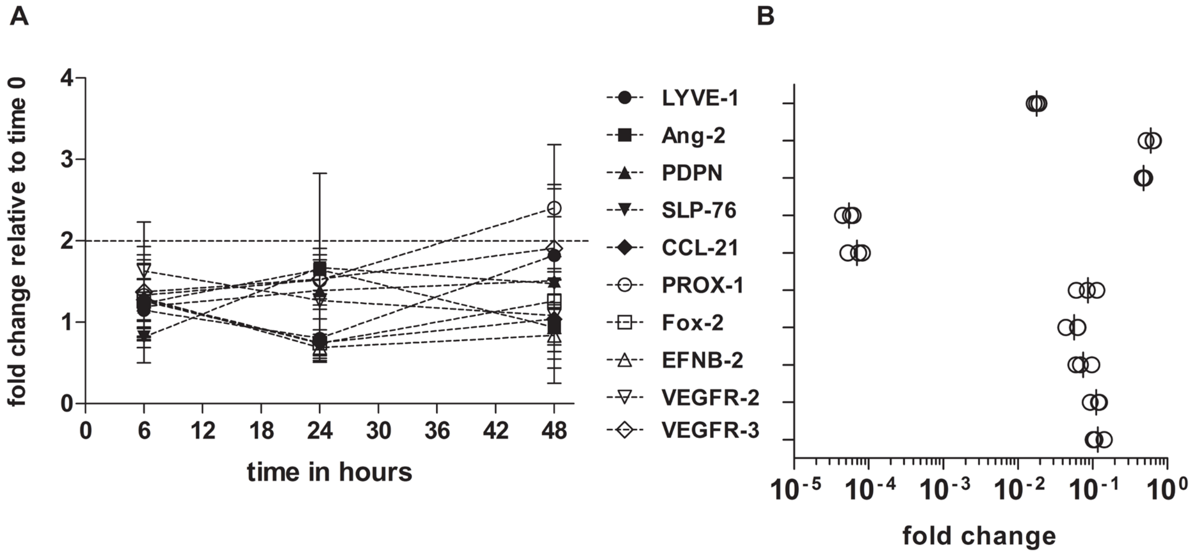

Supplementary Figure 1: A. Constitutive gene expression of MEC-secreting factors and VEGFRs was maintained over a 48 h culture. An arbitrary cut-off was set at 2-fold over the 0-h time point. **B.** Fold change at 24 h of the same factors as compared with CD31 gene expression set to 1 ($2^{-\Delta\Delta Ct}$ method). Two of the factors tested (SLP-76 and CCL-21) were weakly expressed in comparison. All values $\leq 10^{-7}$ were considered undetectable. Error bars represent the mean ± SD of three replicates.

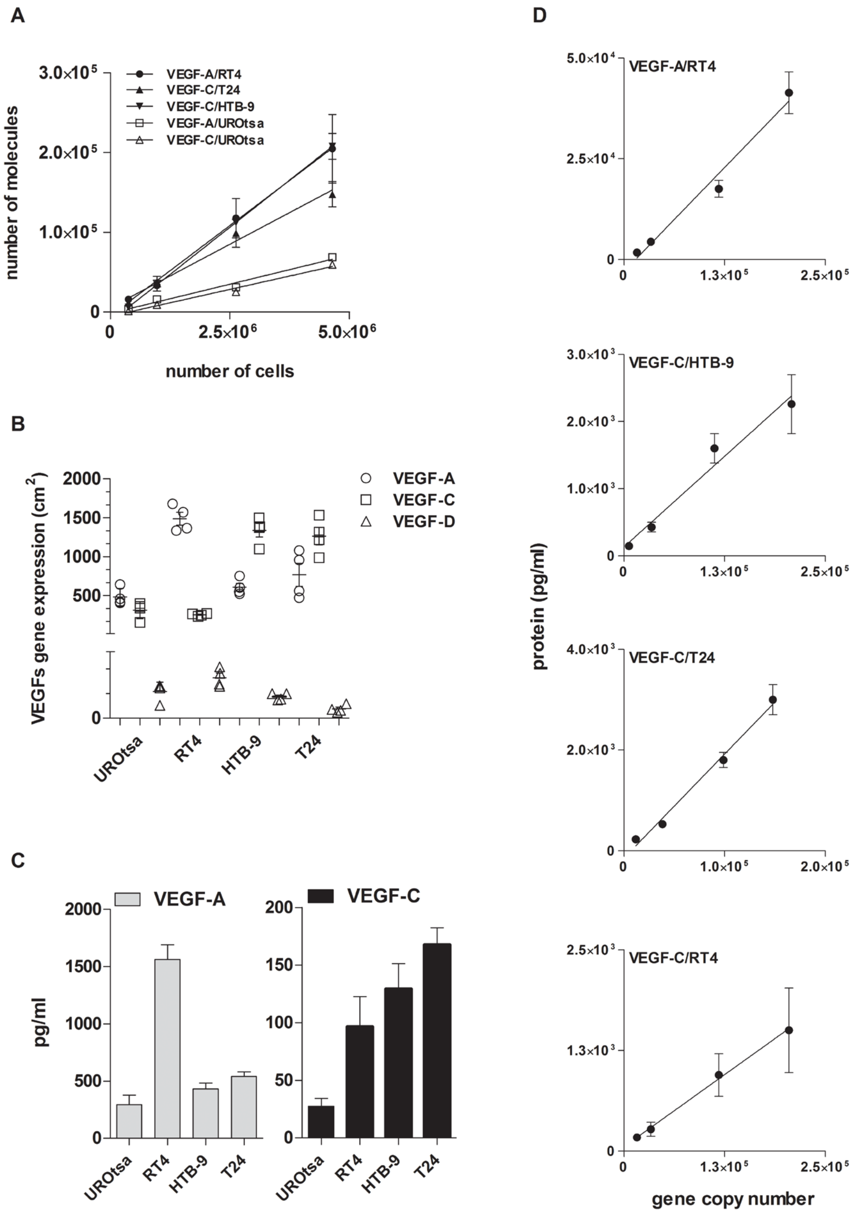

Supplementary Figure 2: ***In vitro*** **analysis of lymphangiogenic growth factors and their modulation. A.** Numbers of VEGF molecules were plotted against the number of cultured cells. RT4 cells produced the highest number of VEGF-A molecules (black circle), compared with cell lines T24 and HTB-9, while both T24 and HTB-9 cell lines produced the highest number of VEGF-C molecules (black triangle and black inverted triangle, respectively), as compared with RT4. Normal urothelium cells (UROTsa) produced both VEGF-A (open square) and VEGF-C (open tringle) to a lesser extent than cancer cells. **B.** The amount of constitutive gene expression upon 72 h of culture of VEGF-A (circle), VEGF-C (square) and VEGF-D (triangle) by three BCa cell lines (RT4, HTB-9, and T24) and uroepithelial cells (UROtsa) was based on the sizes of growth areas (cm^2). Each factor is reported in quadruplicate because of four specific growth areas (150cm^2, 75cm^2, 25cm^2, and 9.6cm^2). An absolute quantification using 1:10 serial dilution standard curves for each gene tested was performed. Two-segment y-axes were considered to show the significant difference in expression of VEGF-A by RT4 ($p < 0.01$; Kruskal-Wallis test) and VEGF-C by HTB-9 and T24 ($p < 0.01$; Kruskal-Wallis test), as compared with the negligible, although significant ($p < 0.05$) expression of VEGF-D by all cell lines studied. **C.** VEGF-A and VEGF-C protein release from the same batches of cells used for gene expression analysis. Each cell line produced amounts of VEGF-A ($p < 0.01$; Kruskal-Wallis test) and VEGF-C ($p < 0.01$; Kruskal-Wallis test) in keeping with gene expression data. Error bars represent the mean ± SD of three replicates **D.** Amounts of VEGF-A proteins from RT4 cells or VEGF-C proteins from HTB-9, T24 or RT4 cells were plotted against respective VEGF-induced gene expression (copy number). These significant linear correlations (VEGF-A/RT4 $r = 0.92$, $p < 0.0001$; VEGF-C/HTB-9 $r = 0.97$, $p < 0.01$; VEGF-C/T24 $r = 0.99$, $p < 0.01$; VEGF-C/RT4 $r = 0.99$, $p < 0.01$; Pearson correlation coefficient) allowed calculation of both VEGF-A and VEGF-C produced by each batch of BCa cells based on their concentration.
